# The Thyroid Forecast: A Case of a Thyroid Storm Diagnosed Under Unforeseen Circumstances

**DOI:** 10.7759/cureus.37707

**Published:** 2023-04-17

**Authors:** Kofi Seffah, Robert Lamptey, Sammudeen Ibrahim, Walter Y Agyeman, Basilio Addo

**Affiliations:** 1 Internal Medicine, Piedmont Athens Regional Medical Center, Athens, USA; 2 Trauma and Emergency, Korle-Bu Teaching Hospital, Accra, GHA; 3 Graduate Medical Education, Piedmont Athens Regional Medical Center, Athens, USA

**Keywords:** wartofsky, burch, risk factors, goiter, chest pain, predictive tools, distributive shock, thyroid, thyroid storm

## Abstract

Hyperthyroidism and thyrotoxicosis are common conditions in clinical practice. Untreated, they are associated with several co-morbidities. One of these conditions, and arguably the most lethal, is the thyroid storm. Our case presentation is one of a young female who had previously been diagnosed with thyroid illness but was lost to follow-up, subsequently given a clean bill of health, and emerged with what would eventually be diagnosed as a thyroid storm. While the thyroid storm may pose some diagnostic challenges, it has come a long way in securing diagnostic tools. What remains is a tool for physicians and patients to stratify patients by their risk of developing a storm in the outpatient setting.

## Introduction

Several medical conditions are described as rare in the context of how often they are encountered relative to other conditions. A closer look at some of these conditions reveals that they occur more frequently than perceived or are often misdiagnosed. Labeling them as rare often paints a distant picture. An example of a condition that continues to carry this "rare" tag but may arguably be more common in practice includes the thyroid storm [1]. The incidence rate is 0.57 to 0.76 persons per 100,000 per year in the general population and 4.8 to 5.6 per 100,000 hospitalized patients per year in the United States [2]. A lot of research has gone into identifying and managing this condition [3,4]. However, there remains a gap in our knowledge regarding the prediction of who is most likely to succumb to this. This case report seeks to explore what this knowledge gap represents in clinical practice and why more attention needs to be paid to this area.

## Case presentation

Our patient was a 29-year-old female who presented to the emergency with complaints of chest pain. She described the pain as severe, crushing, pleuritic central pain that began about two hours prior to her arrival at the emergency. At the point of contact in the emergency, the only significant medical history we had was that she was involved in a motor vehicle accident three weeks prior, with liver and kidney lacerations and a fractured right humerus as a result. Her hospital course following her incident was noted to improve progressively, with the patient ultimately opting to be discharged before medical closure. She spent a total of 18 days on admission following her accident and presented to us five days after her discharge. It must be noted that on discharge, the patient followed up with her primary care provider, who, upon thorough evaluation, provided a clean bill of health about two days before her current presentation.

She noted a low-grade fever accompanying her symptoms. She denied cough or sputum production. The pain was non-radiating, unremitting, non-pulsatile, and intense. This was her first such episode, and she had no other complaint. She had no previously documented cardiovascular risk factors.

On initial evaluation, the patient was noted to have a tachycardia of 180 beats per minute, though normotensive. She was tachypneic at 40 cycles per minute, though saturating at 100% on room air. Body temperature was 99.9° F. Physical examination was otherwise unremarkable, with lungs clear to auscultation and patient mentation remaining stable.

Table 1 summarizes her findings for complete blood count on initial evaluation.

**Table 1:** Complete blood count Findings show normocytic anemia with leukocytosis. While her leukocytosis was initially attributed to sepsis, the normocytic picture was explained by her recent blood loss from the motor vehicle collision.

Test	Value	Reference range
Hemoglobin (g/dL)	9.8	12.0 - 16.0
Packed cell volume (%)	31	35.0 - 46.0
Platelet count (10*3/µL)	476	130 - 400
White cell count (10*3/µL)	18.4	4.00 - 10.50
Mean corpuscular volume (fL)	87.9	78.0 - 100.0

Table 2 shows her blood electrolytes on the initial presentation. 

**Table 2:** Basic metabolic panel Lactate remained within normal limits on arrival, posing another diagnostic challenge. Hypernatremia, hyperchloremia with hypokalemia in the setting of suspected severe sepsis was not deemed unusual, as her renal function was shown to have been affected, with a baseline creatinine of 0.8 and presenting creatinine of 1.69. eGFR - estimated glomerular filtration rate

Test	Value	Reference range
Sodium (mmol/L)	153	133 - 145
Potassium (mmol/L)	3.1	3.3 - 5.1
Chloride (mmol/L)	114	98 - 108
Bicarbonate (mmol/L)	22	22 - 32
Urea nitrogen (mg/dL)	35	6 - 20
Creatinine (mg/dL)	1.69	0.4 - 1.0
Glucose (mg/dL)	123	70 - 100
eGFR (mL/min/1.73sq m)	>60	>60

Table 3 highlights the normal arterial blood gas on presentation. 

**Table 3:** Arterial blood gas Values for her blood gases remained within normal limits, with the oxygen saturation noted to be elevated at 391, likely due to the high flow cannulation she received on arrival. pO_2 _- oxygen saturation; pCO_2 _- partial pressure of carbon dioxide

Test	Value	Reference range
pH	7.42	7.35 - 7.45
pCO_2_ (mmHg)	36.9	35 - 45
pO_2_ (mmHg)	391	80 - 100
Bicarbonate (mmol/L)	23.6	22 - 26
Lactate (mmol/L)	1.6	<2.0

The urine drug screen was positive for cannabinoids.

CT scan of the chest ruled out a pulmonary embolism but showed an interval increase in her heart size and a right-sided pleural effusion.

At this point, given the high white cell count of 18.40* 10^3/µL, recent admission, and surgery with the need for antibiotics for a diagnosed pneumonia, and the request to leave before medical closure, this led us to a diagnosis of severe sepsis. We commenced antibiotics with* Pseudomonas* coverage, vancomycin and piperacillin-tazobactam, as records indicated that she had developed pneumonia during her recent admission, where sputum cultures were positive for this organism. She also received intravenous Ringer's lactate solution at 30 ml per kilogram, in keeping with this diagnosis. Parenteral morphine was given for pain. Due to the severity of her symptoms and pre-empting a poor prognosis with a potential need for airway protection, the intensivist team was consulted.

Her symptoms, however, notably got worse rather rapidly following the initiation of treatment. About an hour into treatment, her tachypnea had not resolved, and the patient was noted to develop a raised jugular venous pulse. Initial high sensitivity troponin came back at 474, and the repeat after two hours was 773.Brain natriuretic peptide (BNP)was noted to be high at 3285.0. Electrocardiogram (EKG) showed sinus tachycardia. Bedside transthoracic echo revealed an ejection fraction of 15% with moderate diastolic dysfunction. Chest pain remained unresponsive to morphine. Vitals remained the same as before; however, at this point, our concern was an impending shock. By this time, results from her thyroid panel returned, as shown in Table 4.

**Table 4:** Thyroid function panel The initial thyroid function panel shows a thyrotoxic picture consistent with what would be found in a hyperfunctioning thyroid. In the setting of severe sepsis, this would pose a hard result to interpret. However, her interval history and subsequent deterioration led us to the diagnosis of a thyroid storm. TSH - thyroid stimulating hormone; T3 - triiodothyronine; T4 - thyroxine

Test	Value	Reference range
TSH (uIU/mL)	<0.030	0.340 - 5.600
Free T4 (ng/dL)	>5.60	0.46 - 1.42
Total T3 (ng/dL)	696.0	87.0 - 178.0

We promptly commenced treatment with methimazole, metoprolol, and intravenous corticosteroids. Interval history from the patient revealed that three years ago, while pregnant, she was informed that her thyroid hormones were deranged, and she developed a goiter. However, following her delivery, she had several visits to her primary care provider but was given a clean bill of health. Her goiter had since resolved. Referring to imaging studies done following her accident three weeks earlier, there was the presence of a heterogeneously enlarged edematous thyroid attributed to shock thyroid at the time. Follow-up was therefore not recommended on these grounds. 

Within 30 minutes of this additional history, our patient coded. She endured 60 minutes of cardiopulmonary resuscitation (CPR), involving several shocks, and ultimately required venoarterial extracorporeal membrane oxygenation (VA ECMO) by cardiothoracic surgery. Additionally, following renal assessment by the nephrology team, the patient was started on continuous renal replacement therapy. Her prognosis remained guarded, and less than 12 hours after coding and with the above interventions commenced, the patient died.

## Discussion

Thyroid storms are defined as rare clinical manifestations of hyperthyroidism, with sympathetic overdrive manifesting as tachycardia, fever, hypotension, and heart failure [5]. Common underlying etiologies include Grave's disease, multinodular goiters, and exposure to medications such as amiodarone and checkpoint inhibitors [5]. Any form of physiological stress, from labor to extremes of exertion and surgeries, may trigger this entity [2]. Notably, the mortality rate is up to 10% [6,7]. Risk factors include poor management of hyperthyroidism and infection [1].

Diagnosis is often made clinically, in the setting of terminal symptoms, including altered mental status, hypotension, atrial fibrillation, and refractory heart failure [8]. The Burch-Wartofsky score aids in the process, with a score of 45 or more indicating a likely storm and with lower scores portending an impending one [8]. The consensus currently is that the level of thyroxine (T4) or triiodothyronine (T3) is not a predictor of the severity of the condition once diagnosed [9].

Treatment, once diagnosed, is multifaceted. Supportive care is key - a thionamide, which blocks further production of the hormone, iodine preparation to prevent the transport of pre-formed hormone into the circulation; a beta-blocker to mitigate the sympathetic effect of the hormone on the body; a steroid to supplement any adrenal insufficiency, address any autoimmune component and reduce the conversion of T4 to T3. Other modalities, including bile acid sequestrants and iodinated radiocontrast agents, have also been reported as suitable for use [5,9].

Our patient was identified to be in a storm about one hour after her arrival and after initial resuscitative methods. She received all treatments recommended for the disease but did not recover following her cardiac arrest. Despite the best level of care, with guidelines directed, and specialist involvement, her condition remained guarded, underscoring the grim prognosis of this disease.

We believe a condition with a mortality of about 10% amongst those diagnosed with hyperthyroidism should not be described as rare. We would like to take this opportunity to call for more research in the area, for the development of a more sensitive predictive or risk stratification tool, for those who are at high risk for a storm. This tool will inform additional pre-emptive outpatient management, regardless of presentation. We believe that this will be an additional step forward for such patients to know what they stand to lose or gain early on in their management. While tools such as the Burch-Wartofsky help make a diagnosis, a condition so fatal will benefit from a more individualized approach early on in the diagnosis of thyroid disease. Factors such as medication exposure, surgeries, stress, pregnancy, and illness in such patients may be developed into prediction tools.

## Conclusions

While we are aware that individuals diagnosed with thyrotoxicosis are always at risk for a thyroid storm, there remains no stratification tool for both physicians and patients to fall on. The current Burch-Wartofsky scale only attempts to affirm if current active symptoms are likely to be from a storm or otherwise. This case report argues that if this patient, previously diagnosed with a thyroid condition, had been given such information at the beginning, including her prognosis, she may probably have taken steps early on, may have been treated differently following her car accident, and then during her recent admission, and may have had a different outcome. Further research is needed in the area to address this gap.
